# Penicillin de-labelling in vancouver, British Columbia, Canada: comparison of approaches, outcomes and future directions

**DOI:** 10.1186/s13223-023-00777-4

**Published:** 2023-04-18

**Authors:** Sujen Saravanabavan, Amneet Aulakh, Josh Douglas, Chelsea Elwood, Stephanie Erdle, Jennifer Grant, Kristopher T. Kang, Natasha Kwan, Katie Lacaria, Tim T. Y. Lau, Colin Lee, Victor Leung, Yu-Chen Lin, Allison Mah, Anne Nguyen, Vanessa Paquette, Ashley Roberts, Melissa Watt, Julie Van Schalkwyk, Bei Yuan Zhang, Raymond Mak, Tiffany Wong

**Affiliations:** 1 Department of Pediatrics, Faculty of Medicine, University of Ottawa, Ottawa, ON Canada; 2 Vancouver General Hospital, Vancouver, BC Canada; 3 Lions Gate Hospital, Vancouver, BC Canada; 4 B.C. Women’s Hospital and Health Centre, Vancouver, BC Canada; 5 BC Children’s Hospital, Vancouver, BC Canada; 6 Providence Health Care, Vancouver, BC Canada; 7 Faculty of Medicine, University of British Columbia, Vancouver, BC Canada; 8 Faculty of Pharmaceutical Sciences, University of British Columbia, Vancouver, BC Canada; 9 Lower Mainland Pharmacy Services, Vancouver, BC Canada

**Keywords:** Penicillin, Drug allergy, De-label, Antimicrobial stewardship

## Abstract

**Background:**

Inaccurate penicillin allergy labels lead to inappropriate antibiotic prescriptions and harmful patient consequences. System-wide efforts are needed to remove incorrect penicillin allergy labels, but more health services research is required on how to best deliver these services.

**Methods:**

Data was extracted from five hospitals in Vancouver, British Columbia, Canada from October 2018-May 2022. The primary outcomes of this study were to outline de-labelling protocol designs, identify the roles of various healthcare professionals in de-labelling protocols and identify rates of de-labelling penicillin allergies and associated adverse events at various institutions. Our secondary outcome was to describe de-labelling rates for special populations, including pediatric, obstetric and immunocompromised subpopulations. To achieve these outcomes, participating institutions provided their de-labelling protocol designs and data on program participants. Protocols were then compared to find common themes and differences. Furthermore, adverse events were reviewed and percentages of patients de-labelled at each institution and in total were calculated.

**Results:**

Protocols demonstrated a high level of variability, including different methods of participant identification, risk-stratification and roles of providers. All protocols used oral and direct oral challenges, heavily involved pharmacists and had physician oversight. Despite the differences, of the 711 patients enrolled in all programs, 697 (98.0%) were de-labelled. There were 9 adverse events (1.3%) with oral challenges with mainly minor symptoms.

**Conclusions:**

Our data demonstrates that de-labelling programs effectively and safely remove penicillin allergy labels, including pediatric, obstetric and immunocompromised patients. Consistent with current literature, most patients with a penicillin allergy label are not allergic. De-labelling programs could benefit from increasing clinician engagement by increasing accessibility of resources to providers, including guidance for de-labelling of special populations.

**Supplementary Information:**

The online version contains supplementary material available at 10.1186/s13223-023-00777-4.

## Background

Globally, 8–25% of patients are identified as penicillin allergic [1–3], but up to 98% of these patients are found to be be penicillin tolerant after an oral challenge [4–7]. Inappropriate penicillin allergy labels result in suboptimal antimicrobial treatment, increased risk of surgical site and resistant organism infections, adverse drug events, and higher healthcare costs [5]. A variety of resources are available to de-label penicillin allergies. Taking a history with clinical tools such as the PEN-FAST score [8] can risk-stratify patients and remove the label if there is a history of tolerating penicillins or the reaction is a side effect. Intradermal penicillin skin tests (PSTs) followed by oral challenges and direct oral challenges (DOCs) without a PST have been utilized [9, 10]. There is also data on de-labelling obstetric [11]and pediatric patients [12] that supports the safety of DOCs in these special populations.

Currently, there remains no standard penicillin allergy de-labelling approach due to emerging data on definitive methodologies, communication barriers between programs and protocol development for special populations [2]. However, there are themes on how to optimize protocols, including collaboration of multidisciplinary teams [13]. Particularly, pharmacist-led programs are safe and effective [14, 15]. Integrating antimicrobial stewardship (AMS) with de-labelling protocols supports de-labelling and AMS practices [16], as does leveraging technology such as electronic medical records (EMR) [17]. In one study, computerized penicillin de-labelling guidelines increased penicillin or cephalosporin use two-fold [18].

Ample data is available on de-labelling within individual practice pathways, focusing on risk stratification, information accuracy and inter-professional communication [13]. In contrast, there is a paucity of data around system-level service delivery and maintaining sustainable practices, creating challenges to implement de-labelling programs. We compare penicillin allergy de-labelling approaches at five hospitals in Vancouver, British Columbia (BC), Canada and their outcomes.

## Methods

### Setting and population

Data was collected from institution-specific databases at five hospitals in Vancouver, BC, Canada. The hospitals and their specific penicillin de-labelling populations were as follows: St. Paul’s Hospital inpatients and outpatients, Vancouver General Hospital (VGH) internal medicine inpatients and leukaemia and bone marrow transplant (LBMT) outpatients, BC Women’s Hospital (BCWH) obstetric patients between 32–36 weeks gestational age, Lion’s Gate Hospital (LGH) inpatients and outpatients, including obstetric patients, and BC Children’s Hospital (BCCH) general pediatric and pediatric oncology inpatients. Dates of data collection varied based on institution but were overall collected from October 2018 to May 2022.

### Data extraction

Participating institutions provided their penicillin allergy de-labelling protocols and data on de-labelling program participants, including target population, program start date, clinical setting, patient identification process and methods of testing. Descriptive data on de-labelling team members, their roles and processes were collected. Participant data for each program included: number of patients enrolled, number approached but not tested (due to patient refusal, medical contraindication, NPO status, or previous severe reactions), number de-labelled on history. The number of participants who had a PST, an oral challenge after a negative PST and a DOC with the result of each test was also recorded. Lastly, data on adverse events was collected.

### Outcomes and data analysis

This primary outcomes of this study are: (1) outline penicillin de-labelling protocol designs, (2) identify the roles of healthcare professionals in different de-labelling protocols and (3) identify rates of de-labelling penicillin allergies and associated adverse events at various institutions. The secondary outcome of our study was to describe de-labelling rates for pediatric, obstetric and immunocompromised subpopulations. Protocols were assessed for common themes and differences. Furthermore, adverse events were reviewed and percentage of de-labelled patients within each program and in total were calculated.

### Ethics

A waiver was granted from the institutions’ research ethics boards due to the quality improvement nature of this project.

## Results

### Demographics

Our data included a large multicenter population, who were predominantly adult (691 patients or 98.0%), non-pregnant (522 patients or 73.4%) and in the outpatient (234 patients or 64.7%) setting. Table 1 summarizes the demographics of patients included in various de-labelling programs.

**Table 1:** Demographics of patients enrolled in penicillin de-labelling protocol

Subgroup	Number (Total = 711)	Percent
Age		
Pediatric (< 18)	20	2.8
Adult (≥ 18)	691	98.0
Inpatient vs. outpatient^1^		
Inpatient	129	35.3
Outpatient	236	64.7
Cancer history^2^		
Yes	3	0.4
No	708	99.6
Obstetric status		
Pregnant	189	26.6
Not Pregnant	522	73.4
Bone marrow transplant patient		
Yes	56	7.9
No	655	92.1

^1^Data does not include LGH and SPH as this data is not closely tracked

^2^Many centres did not have this data available, so this number is underrepresented

### De-labelling protocol designs and healthcare provider roles

**Fig. 1:**
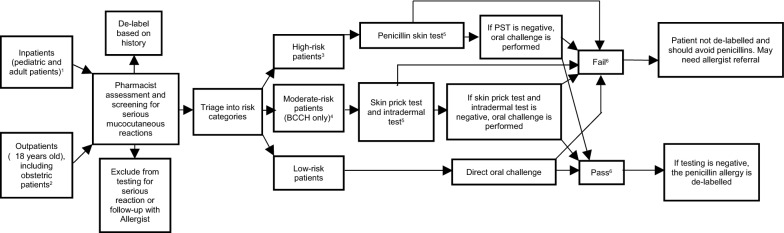
Overview of penicillin de-labelling protocols at various institutions. ^1^Institutions with inpatient programs: SPH, VGH Internal Medicine, LGH, BCCH. ^2^Institutions with outpatient programs: SPH, VGH LBMT Program, BCWH (pregnant patients only), LGH. ^3^LGH’s obstetric de-labelling program does not risk stratify patients, and follows the “high-risk” pathway. ^4^Only BCCH’s protocol has a “moderate-risk” category; all other institutions have only high and low-risk categories. ^5^The reagents used for skin testing vary based on the institution. BCCH, BCWH and LGH use Penicillin G. VGH and SPH test both Penicillin G and a minor determinant mixture. ^6^ “Pass” is defined as having a negative skin test and not reacting to the oral challenge/DOC. In contrast, “fail” is defined as having a positive skin test or having a reaction to the oral challenge/DOC

The various institutions offer inpatient programs, outpatient programs or both. Some institutions have developed de-labelling programs for special populations such as obstetric, immunocompromised, oncology or pediatric patients. Patients are identified through various institution-specific mechanisms and assessed by a healthcare provider. All institutions have their initial assessments by a pharmacist, other than BCWH where the assessment is done by a physician. Based on these assessments, all programs can de-label based on history. Patients are then risk-stratified into high or low-risk categories. BC Children’s Hospital also has a moderate-risk category. The risk stratification tool used varies, so the exact definitions of high and low-risk populations changes based on the institution. In general, high-risk patients are characterized by: how long ago a patient’s penicillin reaction was, having a reaction that was anaphylactic or mucocutaneous in nature, and if treatment was required for the reaction. If patients do not satisfy this criteria or they have taken penicillins again without reacting, patients are low-risk. SPH, VGH’s internal medicine inpatient program and BCWH use the PEN-FAST tool. VGH’s LBMT program and BCCH uses algorithms adapted from the Canadian Paediatric Society (CPS) practice point on beta-lactam allergies [12, 19]. Both programs at LGH use institution-specific protocols adapted from guidelines provided by the BC Provincial Antimicrobial Stewardship Clinical Expert (PACE) committee [20]. Across programs, low-risk patients undergo a DOC, high-risk patients undergo a PST followed by an oral challenge. Moderate risk patients at BCCH have a skin prick and PST performed, followed by an oral challenge. If the DOC or oral challenge is passed, then patients have their penicillin allergy de-labelled. If patients have a positive skin prick or PST, or react to their DOC or oral challenge, then their penicillin allergy is not de-labelled and they may need follow-up with an allergist. Figure 1 provides a visual summary of all the de-labelling protocols, and Table 2 provides a detailed overview of each institution-specific protocol. Further details on each institution’s de-labelling protocol can be found in the Additional file 1.

**Table 2:** Overview of institution-specific de-labelling programs

	St. Paul’s Hospital	Vancouver General Hospital		BC Women’s Hospital	Lion’s Gate Hospital		BC Children’s Hospital
Target Population	All inpatients and outpatients	Internal medicine patients	Leukemia and bone marrow transplant patients	Obstetric patients	Adult inpatients and outpatients	Obstetric patients	Pediatric inpatients and oncology patients
Start Date	Inpatients April 2015; Outpatient April 2017	July 2020	October 2018	July 2020	2018	Sept 2020	November 2019
Inpatient vs. Outpatient	Both	Inpatient	Outpatient	Outpatient	Both	Outpatient	Inpatient
Patient Identified by	Inpatients identified through EMR by pharmacist, nurse practitioners or physicians; Outpatients referred by ID physician	Electronic Medical Record	Pharmacist or transplant nurse navigator	Referral	Inpatient or community referral	Community referral	Pharmacy screen or pediatrics team
Patients Initially Assessed By	Pharmacist	Pharmacist	Pharmacist	Physician	Pharmacist	Pharmacist	Pharmacist
De-label based on history?	Yes	Yes	Yes	Yes	Yes	Yes	Yes
Risk-Stratification Tool	PEN-FAST	PEN-FAST	Algorithm adapted from the (CPS) practice point on beta-lactam allergies	PEN-FAST	Institution-specific protocol based on PACE committee	Institution-specific protocol based on PACE committee	Algorithm adapted from algorithm published in the CPS practice point on beta-lactam allergies
Skin Testing Performed	Yes	Yes	Yes	Yes	Yes	Yes	Yes
Skin test Performed by	Allergist	Allergist	Pharmacist or Allergist	Allergist	Medical Daycare Nurse	Medical Daycare Nurse	Allergist (approval for pharmacist pending)
Direct Oral Challenge Performed	Yes	Yes	Yes	Yes	Yes	Yes (if passed skin test)	Yes
Oral Challenge and DOC Performed By	Allergist or patient’s MRP	Bedside nurse	Daycare unit nurse or Allergist	Usually pharmacist but can be done by anyone on team	Inpatient challenge performed by AMS pharmacist; Outpatient challenge performed by medical daycare nurse	Medical daycare nurse	Pharmacist
Monitoring	Bedside nurse (directed by an allergist, pharmacist or care team physician)	Bedside Nurse	Virtual (by allergist) or In-Person monitoring (by daycare nurse)	Clinic Nurse	Inpatient by AMS pharmacist and bedside nurse; Outpatient by medical daycare nurse or physician	Medical Day Care Nurse	Parent

### Rates of de-labelling and adverse events

All protocols de-labelled at least 95% of patients enrolled in their programs, with an average of 98.0% of patients being de-labelled across all programs. The number of patients who went through the different stages of the de-labelling protocol, including institution-specific rates of de-labelling can be found in Table 3. In total, there were 9 adverse events associated with DOCs or oral challenges, which are summarized in Table 4.

**Table 3:** Outcomes of institution-specific de-labelling protocols

	St. Paul’s Hospital^1^	Vancouver General Hospital		BC Women’s Hospital	Lion’s Gate Hospital	BC Children’s Hospital	Total
		Inpatient Program	Bone Marrow Transplant Program				
Total participants enrolled in penicillin de-labelling program	132	109	56	180	214	20	711
Total participants approached who did not enroll in penicillin de-labelling program	Not available	96	6	0	Not available	Not available	102
De-labelled based on history	40	58	10	6	89	1	204
Penicillin skin testing	75	15	14	41	64	1	210
Negative penicillin skin testing	75	15	14	40	58	1	203
Oral challenge after negative penicillin skin test	72	15^5^	14	40	58	1	200
Adverse reactions with oral challenge after negative skin test	1	1	0	0	1	0	3
Direct oral challenge	17	36	32	126	61	17	289
Adverse events with direct oral challenge	0	0	2	4^2^	0	0	6
Total de-labelled	131	108	54	178	207	19	697
% De-labelled	99.2%	99.1%	96.4%	98.8%	96.7%	95%	98.0%

^1^SPH: Data was only provided for January 1, 2020-January 1, 2021 due to an EMR change in November 2019 making it difficult to extract data

^2^Two patients with a delayed rash and one with nausea, emesis and subjective pruritis. They were de-labelled, but a mild delayed reaction was documented in their chart

**Table 4:** Adverse events with direct oral challenges and oral challenges

Reaction details	Number of Reactions:	Reaction Severity^1^
Urticaria at the time of the DOC or oral challenge	2	Grade 1 reactions
Delayed rash or urticaria	4	Grade 1 reactions
Delayed rash or subjective pruritis, with gastrointestinal symptoms	2	Grade 2 reactions
Flushing and respiratory symptoms	1	Grade 2 or 3 reaction
Total	9	

^1^Reaction severity is graded based on the World Allergy Organization allergic reaction grading system [21]

### Special populations

Programs focused on special populations demonstrated a high rate of penicillin allergy de-labelling. Immunocompromised patients in the LBMT program had a 96.4% rate of de-labelling and pediatric patients demonstrated a 95% rate of de-labelling. 98.8% of obstetric patients enrolled in BCWH’s program had their penicillin allergy de-labelled.

## Discussion

Across programs, 697 of 711 (98.0%) patients with a labelled penicillin allergy were de-labelled. This number is consistent with previous data indicating that most patients with a penicillin allergy label are not allergic [5, 7]. Based on the World Allergy Organization allergic reaction grading system, there were six grade 1 reactions and two grade 2 reactions [21]. The final adverse reaction could be grade 2 or 3 depending on whether the airway symptoms were upper airway (i.e. throat clearing) or lower airway (i.e. bronchoconstriction) symptoms. Overall, there were 9 adverse events, showing that de-labelling programs are safe and effective even in populations with safety concerns around de-labelling (i.e. pediatric, obstetric and immunocompromised patients). These services may mitigate consequences of unverified labels, although our data did not assess subsequent antibiotic selection.

Protocol similarities included a high and low-risk patient triaging and using DOCs or oral challenges as the de-labelling gold-standard, which are approaches that have been well-established in previous reviews [2, 22]. All programs performed PSTs only on high-risk patients. Of the 210 high-risk patients across all centres who underwent a PST, 203 had a negative and 7 had a positive test, contributing to the accumulating body of evidence that oral provocative challenges are safe and effective without skin testing [10, 23, 24]. With DOCs, there were low rates of reactions (1.7%), similar to previous literature [25]. Furthermore, all inpatient and outpatient teams were multidisciplinary. Staicu et al. has previously described the benefits of co-ordinating efforts of a multidisciplinary team to promote de-labelling [26].

There was a high degree of variability between programs, including different methods to identify penicillin-allergic patients, different risk-stratification tools, and different providers administering PSTs, conducting DOCs or oral challenges and monitoring patients. Inpatient programs used varying degrees of EMR and clinician referral to identify penicillin-allergic patients, whereas outpatient programs relied on referrals or pharmacist identification. Consequently, there is a difference in record-keeping amongst programs, with each program extracting different data. Furthermore, there is variability in program uptake even within an institution. For example, 46.8% of eligible VGH inpatients were excluded (52 patients). Other programs such as the VGH LBMT program had 90.3% patient uptake. The difference is likely because LBMT patients have frequent follow up, whereas inpatients tend to have higher turnover or may be medically unstable. Overall, there is a gap in the literature regarding how to design penicillin de-labelling services in a way that is safe, sustainable and effective within health systems [13]Despite program differences, they all demonstrated high de-labelling rates and good safety.

Education and tools should be provided to help clinicians identify patients for de-labelling program referral and even de-label patients within their practices. With many BC health authorities transitioning to a single EMR, there has been institutional pressure to implement provincial standardized protocols. Potential benefits of unifying protocols include avoiding duplication of work, ensuring consistent care, improving AMS, and robust record-keeping. Barriers to implementing a provincial and national de-labelling strategy include engaging providers in the de-labelling process and targeting a diverse patient population whose medical needs may vary. However, our data suggests that despite protocol heterogeneity, de-labelling is effective. Perhaps health authorities may focus on education and tool dissemination to encourage clinicians to refer to de-labelling programs and even de-label patients within their practices. If standardization were to occur, greater oversight of centralized organizations, such as the PACE committee would be helpful to address stakeholder concerns. Organizations should also draft best practice guidelines to support prescribers in conducting allergy assessments and oral challenges in low-risk patients. To address this need, www.dropthelabel.ca was created by a multi-disciplinary  group of providers, including allergists, pediatricians, pharmacists, family physicians and other healthcare providers across various institutions in British Columbia to centralize resources, handouts and instructional videos for institutions and caregivers. These resources were created using currently published literature and experience of clinicians with expertise in penicillin allergies. Furthermore, mobile, point of care risk assessment tool adapted from published guidelines [19, 27] has also been created: https://app.firstline.org/en/clients/39-bc-womens-hospital/steps/40356. Notably, other risk-stratification tools used at the institutions in this study include the PEN-FAST tool [8] and institution-specific protocols adapted from PACE committee guidelines [20]. There are continued quality improvement initiatives to ensure de-labelling protocols and system processes are meeting needs of patients over time.

### Limitations

This study has limitations impacting its generalizability. The data was collected retrospectively, and as a result, there was some missing data particularly around adverse events. Additionally, due to a lack of a unified database, data extraction varied between institutions. As data was collected exclusively from Vancouver, it may be difficult to apply to other contexts. Furthermore, these protocols may not be feasible by a community physician as this data was collected from hospital-based institutions where interdisciplinary teams are accessible. Since co-morbidity data was not collected it is unclear how these protocols apply to special populations. Lastly, we were unable to determine the impact of de-labelling on actual penicillin use reduction.

## Conclusions

In conclusion, we assessed de-labelling approaches in terms of the rates of de-labelling, protocol design and roles within multidisciplinary teams. Despite various protocols having a greater than 96% de-labelling rate, there continues to be opportunities to increase clinician engagement by dissemination of de-labelling resources. Future directions should involve more health system research on delivery of national penicillin de-labelling programs and translating that research into optimized de-labelling programs accessible to patients and providers in the hospital and community.

## Supplementary Information

**Additional file 1.** (1) a detailed description of the de-labelling process at each institution (2) the outcomes of the de-labelling process at each instution, including the number of patients enrolled, the number of patients de-labelled and details of the various adverse events.

## Data Availability

All data generated or analysed during this study are included in this published article.

## Abbreviations

PST: Intradermal penicillin skin tests
DOC: Director oral challenge
EMR: Electronic medical record
AMS: Antimicrobial stewardship
BC: British Columbia
NPO: Nil per Os
VGH: Vancouver general hospital
LBMT: Leukemia and bone marrow transplant
CPS: Canadian Paediatric Society
BCWH: BC Women’s Hospital
LGH: Lion’s Gate Hospital
