# Supplementary material for: Penicillin de-labelling in vancouver, British Columbia, Canada: comparison of approaches, outcomes and future directions
Source: Allergy Asthma Clin Immunol. 2023 Apr 18;19:30. doi: 10.1186/s13223-023-00777-4 (PMC10114447; doi:10.1186/s13223-023-00777-4)
Supplement: Supplementary file 1 — Additional file 1. (1) a detailed description of the de-labelling process at each institution (2) the outcomes of the de-labelling process at each instution, including the number of patients enrolled, the number of patients de-labelled and details of the various adverse events. [file 13223_2023_777_MOESM1_ESM.docx]

**Additional file informations:**

Process:

*St. Paul’s Hospital:*

St. Paul’s Hospital runs an inpatient and outpatient penicillin de-labelling program. The outpatient program relies on clinician referrals. Physicians, nurse practitioners or pharmacists identify inpatients through the EMR. Since 2021, risk stratification was done by a pharmacist using PEN-FAST to identify patients a high-risk, low-risk, or very low-risk. If low-risk, an allergist performs a DOC. If high-risk, an allergist performs a PST and a follow-up oral challenge. If very low-risk, the care team may perform a DOC without direct allergist involvement, usually with a pharmacist or infectious disease specialist. All monitoring is done by a nurse.

*Vancouver General Hospital (VGH): Internal Medicine Inpatient Program and Leukemia and Bone Marrow Transplant (LBMT) Program*

VGH’s first penicillin de-labelling program focuses on internal medicine inpatients. A pharmacist identifies inpatients with a penicillin allergy label through the EMR. After a pharmacist assessment using the PEN-FAST tool, patients can be de-labelled based on history or triaged into high or low-risk categories. High-risk patients undergo a PST with a follow-up oral challenge and low-risk patients undergo a DOC. PSTs are performed by an allergist. Oral challenges and DOCs are administered and monitored by nurses.

VGH’s second penicillin allergy de-labelling program is for patients awaiting hematopoietic stem cell transplantation. A pharmacist or nurse identifies patients from their medical record. Initial assessments are conducted by a pharmacist using a questionnaire. If the patient is not de-labelled based on history, they are triaged into high or low-risk categories using a clinical algorithm adapted from the Canadian Paediatric Society (CPS) practice point on beta-lactam allergies (18). High-risk patients are referred to an allergist for further assessment. Low-risk patients undergo a DOC. PSTs are performed by a pharmacist or allergist with follow-up oral challenges performed by a nurse. Non-urgent DOCs are administered in-person or virtually by the allergist. Urgent DOCs may be done without allergist involvement in the daycare unit.

*BC Women’s Hospital (BCWH): Obstetric Patients between 32-36 Weeks Gestational Age*

The BCWH outpatient penicillin allergy de-labelling program is for obstetric patients. Pregnant patients are identified through outpatient referral and triaged by gestational age. An allergist conducts a history. If the patient is not de-labelled on history, the patient is triaged as high or low-risk using the PEN-FAST score. If low-risk, the patient undergoes a DOC. If high-risk, a PST is done by an allergist, followed by an oral challenge if negative. DOCs and oral challenges are usually performed by the pharmacist and monitored by a nurse, under allergist and obstetrician supervision.

*Lions Gate Hospital (LGH): Adult Inpatient/Outpatient and Obstetric Program*

LGH’s first program is for non-pregnant adult patients with a suspected penicillin allergy identified as an inpatient or through outpatient referral. An AMS pharmacist reviews the patient profile, conducts an interview, and risk-stratifies them for a PST or DOC if eligible for testing. Risk-stratification is done through a protocol based on guidance from the BC Provincial Antimicrobial Stewardship Clinical Expert (PACE) committee (19). A nurse performs PSTs for both inpatients and outpatients in an outpatient setting. If the PST is negative, the patient undergoes an oral challenge. Outpatient oral challenges are administered by a nurse. Inpatient oral challenges are administered and monitored by an AMS pharmacist. The infectious disease physician assesses PSTs, oversees the process in the medical daycare and is made aware of inpatient DOCs.

The second de-labelling program de-labels obstetric patients between 32- and 36-weeks gestational age. Patients may be referred as early as first trimester. This program relies on outpatient referral. After an AMS pharmacist reviews the patient profile and conducts an interview, if further testing is necessary, patients undergo a PST by a nurse. If the PST is negative, patients undergo an oral challenge. The infectious disease physician assesses the PST and oversees the process.

*BC Children’s Hospital (BCCH):*

The BCCH de-labelling program is for general pediatric and oncology inpatients identified by the pharmacist or inpatient team. The protocol uses an electronic algorithm published in the CPS practice point on beta-lactam allergies (11,18). A pharmacist assesses the patient with a questionnaire and reviews with the allergist to determine their risk category. If low-risk, a DOC is performed. If moderate-risk, a skin prick and PST is completed by an allergist. If negative, patients undergo oral challenge. Oral challenges are monitored by parents. If skin testing or PST is positive or they are high-risk, they are re-assessed in the outpatient allergy clinic.

Outcomes:

*St. Paul’s Hospital:*

From January 1, 2020-January 1, 2021, 132 patients were labelled with penicillin allergies. There were 75 PSTs and 89 amoxicillin challenges. There was one inpatient who experienced delayed urticaria with gastrointestinal symptoms after passing the initial PST and oral challenge. The total number of patients who were de-labeled for the entirety of the program was not recorded.

*Vancouver General Hospital: Internal Medicine Inpatient Program and Leukemia and Bone Marrow Transplant Program*

Of the 205 internal medicine inpatients identified between July 2020-March 2021 with a penicillin allergy, 96 were not tested due to medical contraindication (27), patient refusal or discharge (52), or other reasons (17). Three patients were not tested due to a history of allergic interstitial nephritis and 1 was not tested due to a history of drug reaction with eosinophilia and systemic symptoms. Of the 109 enrolled, 108 (99.1%) were de-labelled. Twenty patients had a PST prior to an oral challenge, all of which were negative. One patient developed hives during an oral challenge after a negative PST.

Of the 62 LBMT patients screened between October 1, 2018 to December 31, 2020, 1 had a true allergy based on history, 10 were de-labelled on history, and 5 did not consent to testing. All 14 PSTs and follow-up oral challenges were negative. Of the 32 DOCs, 30 were negative and 2 developed a delayed rash. In total, 54 (87.1%) patients were de-labelled.

*BC Women’s Hospital:*

Of the 180 identified patients between July 2019 and April 2021, 178 (98.9%) were de-labelled. Six patients were de-labelled based on history, 40 with a PST followed by a negative oral challenge and 123 with a negative DOC. One patient did not have an oral challenge due to an equivocal skin test and there were four reactions to DOCs. Three patients had minor reactions: two developed a delayed, non-mucocutaneous rash and one had a delayed reaction with 3 episodes of emesis, one week of nausea and subjective pruritus. These three patients were de-labelled. The last patient developed a morbilliform rash with no mucocutaneous features and was not de-labelled.

*Lions Gate Hospital: Adult Inpatient and Outpatient Program, including Obstetric Patients*

Between both programs, 207 (96.7%) of the 214 patients tested were de-labelled between June 2018 and April 8, 2021. Data was not collected on the total number of patients assessed. Eighty-nine patients were de-labelled based on history, 32 from an outpatient DOC, 29 from an inpatient DOC and 57 by PST followed by an oral challenge. Between September 2020 and April 8, 2021, 9 pregnant patients were de-labelled. In total, 7 patients were confirmed to be allergic. Six had a positive PST and 1 had a negative PST but a positive oral challenge.

*BC Children’s Hospital:*

20 patients were assessed, but one was not de-labelled due to a hospital transfer before the DOC. All 19 patients evaluated through this program from November 2019 to May 2022 were de-labelled. One was de-labelled on history, 1 had skin testing followed by a negative oral challenge, and 17 had negative DOCs. There were no adverse reactions.
